# Neurobiological Effects of Transcranial Direct Current Stimulation over the Inferior Frontal Gyrus: A Systematic Review on Cognitive Enhancement in Healthy and Neurological Adults

**DOI:** 10.3390/biomedicines12061146

**Published:** 2024-05-22

**Authors:** Raffaele Di Fuccio, Anna Lardone, Mariagiovanna De Luca, Leila Ali, Pierpaolo Limone, Paola Marangolo

**Affiliations:** 1 Department of Psychology and Educational Sciences, Telematic University of Pegaso, Piazza dei Santi Apostoli 49, 00187 Rome, Italy; raffaele.difuccio@unipegaso.it (R.D.F.); leila.ali@unicam.it (L.A.); pierpaolo.limone@unipegaso.it (P.L.); 2 Department of Humanities Studies, University Federico II, Via Porta di Massa 1, 80133 Naples, Italy; anna.lardone@gmail.com (A.L.); mariagiovannadeluca1997@gmail.com (M.D.L.)

**Keywords:** inferior frontal gyrus, tDCS, neuromodulation, neuroimaging, electrophysiology, cognitive training

## Abstract

The neurobiological effects of transcranial direct current stimulation (tDCS) have still not been unequivocally clarified. Some studies have suggested that the application of tDCS over the inferior frontal gyrus (IFG) enhances different aspects of cognition in healthy and neurological individuals, exerting neural changes over the target area and its neural surroundings. In this systematic review, randomized sham-controlled trials in healthy and neurological adults were selected through a database search to explore whether tDCS over the IFG combined with cognitive training modulates functional connectivity or neural changes. Twenty studies were finally included, among which twelve measured tDCS effects through functional magnetic resonance (fMRI), two through functional near-infrared spectroscopy (fNIRS), and six through electroencephalography (EEG). Due to the high heterogeneity observed across studies, data were qualitatively described and compared to assess reliability. Overall, studies that combined fMRI and tDCS showed widespread changes in functional connectivity at both local and distant brain regions. The findings also suggested that tDCS may also modulate electrophysiological changes underlying the targeted area. However, these outcomes were not always accompanied by corresponding significant behavioral results. This work raises the question concerning the general efficacy of tDCS, the implications of which extend to the steadily increasing tDCS literature.

## 1. Introduction

Over the last decades, numerous studies have explored the impact of non-invasive brain stimulation (NIBS), such as transcranial direct current stimulation (tDCS), to enhance cognitive functions in healthy and neurological populations [1,2,3,4]. Through the application of a weak electrical current to the brain, tDCS modifies cortical excitability, leading to observable behavioral changes [3,5,6,7]. The standard setup involves placing an active electrode (typically sized between 5 × 7 and 5 × 5) over the target area and a “return” electrode on the contralateral supraorbital region or in a place situated away from the head (e.g., contralateral shoulder) [3,5,6,7,8]. Both electrodes interact to create an electric field that alters cell membrane potentials to increase the likelihood of spontaneous neuronal firing. Depending on several factors (e.g., intensity of the current, distance between the electrodes, size of electrodes), the targeted neurons are most likely to depolarize (increase excitability) under anodal tDCS, or hyperpolarize (decrease excitability), under cathodal tDCS, the resting membrane potentials [9]. Some studies have also attempted to leverage the actions of both electrodes synergistically. Studies employing bi-hemispheric montages are grounded on the assumption that by simultaneously stimulating homotopic regions with an anode and a cathode, the interhemispheric balance will shift towards the anode. This, in turn, may potentially favor the cognitive processing carried out on that hemisphere [10,11].

Since tDCS interacts with ongoing neural activity in the brain, to potentiate its efficacy, to date, several protocols have combined tDCS with a cognitive treatment [3,6,7,8]. When safety guidelines are followed, tDCS is considered a safe technique with no significant adverse effects [12,13,14]. The mechanisms underlying the long-term effects of tDCS are different from those during active stimulation. During active stimulation, tDCS is thought to modulate resting membrane potentials, whereas long-lasting effects are thought to depend on the induction of neurotransmitters (e.g., NMDA-R; N-methyl-D-aspartate receptor) through the process of long-term potentiation (LTP) [15]. LTP contributes to neuroplasticity by increasing the connectivity of neurons, which is important for learning and memory processes [16,17,18]. Although tDCS has gained popularity over the last decades as an exciting adjunctive approach for cognitive enhancement in healthy and neurological populations [3,5,6,7], the diversity of the parameters used, and the different characteristics of the targeted samples have generated heterogeneous and controversial results [19,20,21,22]. For this reason, one important aspect to consider is the area being stimulated. Researchers have explored various brain regions as potential targets for tDCS to optimize its effects in clinical applications [19,20]. For example, targeting the left inferior frontal gyrus (IFG) with tDCS has been shown to be effective in enhancing different aspects of language processing [7]. Similarly, stimulating other regions of the brain has yielded different effects on their respective underlying cognitive functions [23]. Overall, identifying the optimal target area for tDCS involves a systematic approach that integrates knowledge of cognitive functions with their underlying neural substrates. For this reason, more recently, researchers have used techniques such as neuroimaging and electrophysiology to optimize the parameters of tDCS for maximal efficacy.

To further delineate the cognitive benefits of tDCS in healthy and neurological populations, after an overview of the most significant behavioral results achieved by applying tDCS over the IFG, our systematic review focused only on tDCS studies that stimulated either the left or the right IFG, combining behavioral training with neuroimaging or electrophysiology. Indeed, the IFG, traditionally considered a key region for language production and, particularly, for speech articulation [24,25], has been subsequently ascribed to different cognitive domains [26,27,28,29,30,31,32,33,34]. 

### 1.1. The Role of the Inferior Frontal Gyrus (IFG) in Cognitive Performance: Behavioral Evidence from tDCS Studies

Due to its crucial role in motor and language control, over the years, the IFG has gained considerable attention from researchers in the tDCS field. Indeed, together with a growing body of tDCS studies further confirming its prominent role in language tasks [7], some tDCS studies have also emphasized IFG involvement in other cognitive domains such as response inhibition, decision-making, working memory processes, and creative thinking [35,36,37,38]. Indeed, by applying bi-hemispheric tDCS with the anode over the right and the cathode over the left IFG in a group of healthy subjects, Leite et al. [36] found an improvement in inhibitory control. Similarly, using the same bi-hemispheric montage, Hertenstein et al. [35] reported an increase in creative thinking and shifting ability (see also Kalil et al. [39]) in the active condition. Zhu et al. [38] examined whether anodal tDCS over the left IFG affects working memory updating while subjects were asked to complete both the visual and auditory letter 3-back tasks. An improvement in response efficiency was observed after stimulation in both tasks. 

Several researchers have also shown that modulation of activity in the left IFG influences language-related processes such as phonological, semantic, and syntactic processing [40,41], discourse production [42], and abstract and concrete word learning [43]. Thus, the left IFG has been considered as the optimal stimulation site for enhancing language processing in healthy speakers. Moreover, the application of tDCS over the left IFG has been shown to be beneficial in language rehabilitation interventions for adult speakers with acquired language impairments [5,7]. Similarly, by targeting the left IFG, tDCS effects, primarily in the language domain, have been documented in neurodegenerative populations such as Alzheimer’s disease, mild cognitive impairment (MCI), and primary progressive aphasia (PPA) [44,45]. In a very recent study by Heimann et al. [46], the effects of anodal tDCS over the left IFG were measured in a group of healthy elderly under 67 years old (YG), a group of healthy elderly aged 68 years and older (OG), and an MCI group by comparing performance in phonological and semantic word fluency tasks before and after 3 days of tDCS. Half of the experimental participants received sham stimulation. Anodal tDCS was associated with higher scores in phonological but not semantic word fluency in both the YG and OG groups. In MCI patients, no differences were observed between the tDCS and sham groups in either word fluency task. Thus, although phonological word fluency can be increased through anodal tDCS in healthy elderly individuals, when cognitive decline has reached a certain stage, as is the case with MCI, this paradigm does not seem to be effective [46].

Indeed, despite the positive results reported in the above studies, less conclusive data have been reached by recent meta-analyses and systematic reviews. In a recent study with anodal tDCS applied over the prefrontal regions, including (but not only) both the right and left IFG, De Boer et al. [1] reported small positive effects in selective attention tasks and no effects on working memory performance and in cognitive flexibility [1]. Faraht et al. [47] conducted a thorough search of the existing literature to identify relevant meta-analyses and systematic reviews on the cognitive effects of prefrontal tDCS for healthy and neuropsychiatric disorders [47]. Significant tDCS effects were found only in sixteen comparisons, among which thirteen had either low or very low quality, while thirty-eight of the remaining thirty-nine comparisons did not report significant effects. Majdi et al. [48] synthesized findings from multiple studies to assess the overall effect of tDCS on cognitive functions in patients with Alzheimer’s disease. Although the authors concluded that tDCS holds promise as a potential intervention in the Alzheimer’s population, they also highlighted the limitations of the existing literature, such as variability in study methodologies and the need for larger, well-controlled clinical trials to establish tDCS efficacy for Alzheimer’s disease [48]. Very similar conclusions were reached by Siegert et al. [49] in a systematic review exploring the effects of tDCS on various cognitive domains, such as attention, memory, executive function, and language in elderly populations. Another meta-analysis aimed at measuring the impact of tDCS on word reading and picture naming failed to observe relevant tDCS effects even when administered over the IFG [50]. Similarly, a recent review by Elsner and colleagues concluded that there is limited evidence that tDCS is effective in improving noun naming at the end of the intervention period and possibly also at follow-up, and the results on its efficacy on verb naming are inconclusive [5]. Nevertheless, it should be noted that an important limitation of these reviews lies in their inclusion of studies in which various frontal regions were targeted with tDCS, neglecting to account for the specific action of tDCS over a specific area, such as the IFG.

### 1.2. tDCS and Neurophysiological Measurements

To further clarify the impact of tDCS effects on different brain regions, and, in particular, over the IFG, several works to date have combined tDCS with neuroimaging techniques such as functional magnetic resonance imaging (fMRI) and resting state fMRI (rs-fMRI) to explore functional neural changes either under an active condition in which subjects are asked, inside the scanner, to perform a cognitive training (i.e., fMRI) [51] or while subjects do not have to commit their cognitive resources towards a specific task (i.e., rs-fMRI) [52,53]. More recently, functional near-infrared spectroscopy (fNIRS) has also been employed to detect the effects of tDCS on hemodynamic activity by measuring task-related changes in oxy-(HbO) and deoxy-hemoglobin (HbR) concentration [54]. Electrophysiology (electroencephalography—EEG, magnetoencephalography—MEG, event-related potentials—ERPs) has also been employed to provide temporal information for optimizing tDCS application [54]. However, the results obtained on this side are also controversial [55]. One of the reasons for such confusion, as previously stated, is related to the fact that in most of the published investigations [55], authors included tDCS studies targeting different prefrontal regions; thus, it is difficult to conclude whether the stimulation was effective over a specific area or not.

As far as we know, to date, no systematic reviews specifically assessing the impact of tDCS over the IFG have been published. Given the amount of behavioral data on this topic, we believe that conducting research in this direction could provide valuable insights in further understanding the role of the IFG in cognitive performance.

In the present review, we systematically analyzed all studies that combined tDCS with neuroimaging (fMRI, rs-fMRI, fNIRS) or electrophysiological measures (EEG, MEG, ERPs) targeting the IFG. Our study specifically included only published papers in which tDCS was used as an adjuvant device for cognitive training, comparing its effects with a sham condition. Indeed, to gain a clear understanding of the role played by tDCS in cognitive performance, it is crucial to also measure the consequences of the cognitive treatment itself when combined with a placebo condition.

## 2. Materials and Methods

### 2.1. Search Strategy and Selection Criteria

We conducted this study according to the protocol registered in protocols.io (ID:98177) using the methodological framework for systematic reviews following the PRISMA 2020 guidelines [56] to ensure comprehensive and transparent reporting of our methods and findings. We systematically searched for articles until February 2024 on three databases, PubMed, Scopus, and Science Direct, to identify relevant publications combining tDCS over the IFG (either the left or the right) with task-related neuroimaging (fMRI, fNIRS) or electrophysiological techniques (MEG, EEG, ERPs) in healthy and neurological populations. The following keywords and Boolean search terms were used: (1) (transcranial direct current stimulation, tDCS) AND (functional magnetic resonance imaging, fMRI) AND (inferior frontal gyrus, IFG) OR (Broca’s area) AND (cognitive performance) OR (cognitive outcomes); (2) (transcranial direct current stimulation, tDCS) AND (resting-state functional magnetic resonance, rs-fMRI) AND (inferior frontal gyrus, IFG) OR (Broca’s area) AND (cognitive performance) OR (cognitive outcomes); (3) (transcranial direct current stimulation, tDCS) AND (functional near infrared spectroscopy, fNIRS) AND (inferior frontal gyrus, IFG) OR (Broca’s area) AND (cognitive performance) OR (cognitive outcomes); (4) (transcranial direct current stimulation, tDCS) AND (electroencephalography, EEG) AND (inferior frontal gyrus, IFG) OR (Broca’s area) AND (cognitive performance) OR (cognitive outcomes); (5) (transcranial direct current stimulation, tDCS) AND (event-related potentials-ERPs) AND (inferior frontal gyrus, IFG) OR (Broca’s area) AND (cognitive performance) OR (cognitive outcomes); (transcranial direct current stimulation, tDCS) AND (magnetoencephalography, MEG) AND (inferior frontal gyrus, IFG) OR (Broca’s area) AND (cognitive performance) OR (cognitive outcomes).

Included articles met the following criteria: (i) only studies that involved the use of tDCS (anodal, cathodal, or bilateral) over the left and/or right IFG; (ii) only studies conducted with participants over 18 years of age; (iii) only studies including an active and a sham condition; (iv) only studies in which tDCS was combined with cognitive training. Articles were excluded if they were reviews or meta-analyses, single case studies, or case series. After eliminating duplicates, all potentially relevant full texts were screened by the authors independently of one another to exclude non-eligible items.

### 2.2. Data Extraction and Analysis

**Figure 1:**
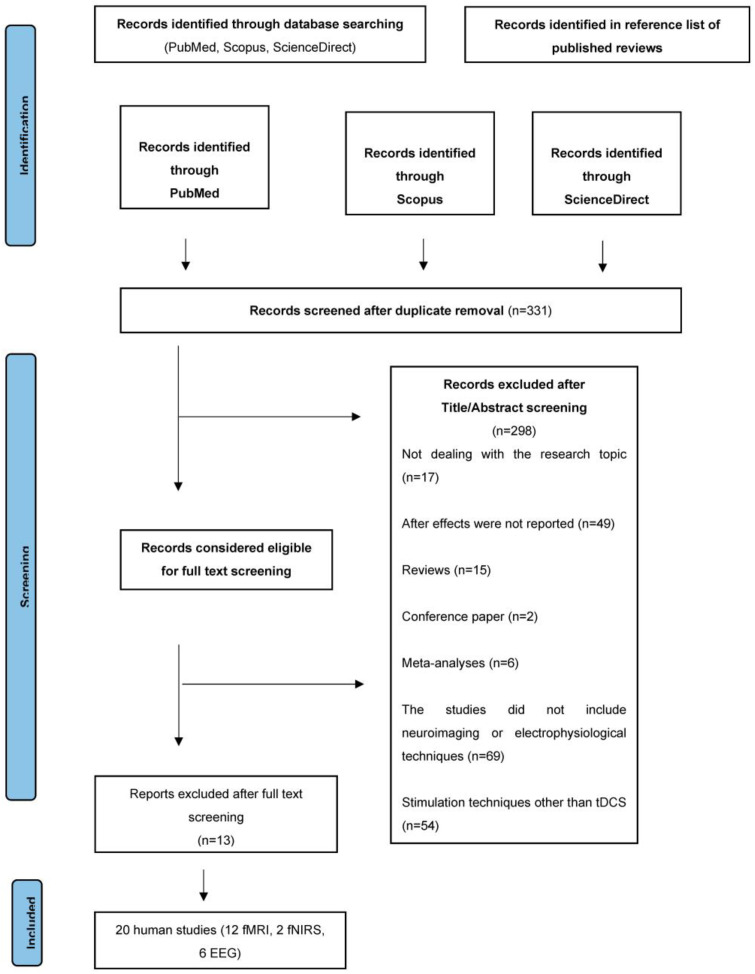
PRISMA diagram illustrating the systematic review process depicting the flow of study selection according to PRISMA guidelines.

A total of 324 articles were retrieved through database searching. Another 45 articles were found thanks to references in published reviews. After the removal of 38 duplicates, a total of 331 articles remained, out of which 17 articles were excluded by title or abstract for not dealing with our research topic, 49 were removed as after-effects were not reported, 15 studies were excluded as referring to reviews, 2 articles were excluded because they were conference papers, 6 papers were excluded as they were meta-analyses, 69 studies did not include the use of neuroimaging or electrophysiological techniques, 54 studies were excluded because they involved stimulation techniques other than tDCS, 11 studies because they involved paediatric or adolescent populations, and 75 studies because they took into consideration a target area other than the IFG. A total of 33 articles were considered eligible for the study. After full-text screening, another 13 articles were removed (see Figure 1).

The selected 20 articles were rearranged according to the characteristics of the studies. fMRI (N = 12) and fNIRS (N = 2) studies were evaluated separately from EEG studies (N = 6) and were further subdivided by reference population such as healthy and neurological patients. To facilitate comparability, we decided to consider within-subject experimental design in studies with healthy participants, even in works that also involved between-subject design [57] (see Table 1 and Table 2).

## 3. Results

The results obtained in the present review are shown in Table 1 and Table 2. Table 1 summarizes neuroimaging studies in healthy and neurological populations. Among these studies, nine were conducted on healthy populations [11,37,58,59,60,61,62,63,64].

The studies by De Rosa et al. [58] and Ehlis et al. [59] used fNIRS with tDCS over the left IFG combined with a visual–spatial working memory task (WM) [59] or a verbal fluency task [55], respectively. In De Rosa et al.’s study [58], faster responses in the behavioral task were accompanied by an increase in hemodynamic activity in bilateral frontal regions in the active condition compared to sham [58]. In Ehlis et al. [59], neither anodal nor cathodal tDCS over the left IFG modulated verbal fluency. However, anodal tDCS increased prefrontal activity during the task.

Four fMRI studies applied anodal tDCS over the left IFG combined with language training [60,61,62,63]. In the study by Fiori et al. [60], anodal tDCS significantly decreased task-related activity at the stimulated left IFG and in the right homolog and between the left IFG and the right insula. The individual decrease in connectivity was positively correlated with improvement in the verb learning task during A-tDCS. In the study by Holland et al. [61], faster naming responses were associated with a BOLD signal decrease in the left IFG but only in the anodal tDCS condition. In the two studies by Meinzer et al. [62,63], the improvement observed in the semantic generation task after anodal tDCS was accompanied by reduced activity in the left IFG [62,63] and in the right middle frontal gyrus (MFG) [62]. In Meinzer et al. [63], the cluster with the strongest tDCS-induced increase was found in the LIFG and anterior insula. Additional significant clusters were found in the bilateral inferior parietal, dorsolateral, medial prefrontal regions, and in the left middle temporal gyrus [63]. In the study by Nissim et al. [64], the authors applied bi-hemispheric tDCS with the anode over the left IFG and the cathode over the RIFG combined with a working memory task. No behavioral differences were found between the real and the sham condition, but the real condition produced a significant increase in functional connectivity between the left ventrolateral and the left dorsolateral prefrontal cortex corresponding to the memory network [64].

Two fMRI works stimulated the right IFG either with anodal tDCS [11,37] or cathodal tDCS [37]. Sandrini et al. [11] applied anodal tDCS over the right IFG together with a response inhibition task (SSRT). Facilitation in response inhibition was reported, which was accompanied by functional connectivity changes in fronto-basal ganglia as well as in the right dorsolateral prefrontal cortex and the right inferior parietal cortex as an integral part of the response inhibition network [11]. In the study by Li et al. [37], no changes were observed at the behavioral level in a choice reaction time task, and only cathodal stimulation over the right IFG accentuated the within-default mode network connectivity.

In addition to the studies with healthy participants, five tDCS studies were conducted on different neurological populations. Specifically, two studies included mild cognitive impairment (MCI) patients with anodal tDCS applied over the left IFG combined with executive function training [65] or a semantic word retrieval task [57]. In the Das et al. study [65], a significantly larger increase in cerebral blood flow (CBF) was observed in the right MFG in the anodal tDCS compared to the sham condition, although no changes were observed at the behavioral level. Meinzer et al. [57] reported a greater improvement in semantic fluency in the anodal tDCS condition compared to sham and, as in the two studies with healthy participants [62,63], reduced activity was observed in the left and right IFG, in the left dorsolateral prefrontal cortex, and in the right MFG. In the two studies involving patients with primary progressive aphasia (PPA), anodal tDCS was applied over the left IFG and combined with written language training [66,67]. In Ficek et al. [66], both the tDCS and sham groups showed significant improvements in the percentage of written correct letters after the treatment. These effects were associated with significant connectivity changes in the left IFG and in the left inferior temporal gyrus [66]. Tao et al. [67] reported a significant increase in behavioral outcomes (percentage of letter accuracy) in the active condition compared to sham, which was maintained at 2-month follow-up. This behavioral evidence was supported by a significant decrease in overall connectivity only in the tDCS group. Specifically, the decrease was due to reduced connectivity between the LIFG and regions outside the peri-sylvian language area in both the left and right hemispheres [67].

Finally, a single study that applied bi-hemispheric tDCS with opposite current over the left and the right IFG (anodal vs. cathodal tDCS, respectively) was combined with a repetition task and involved a group of nonfluent aphasics [10]. Only after the active condition, the mean percentage of correctly articulated syllables and words significantly improved. Interestingly, this improvement positively correlated with increased activity in a cerebral network that includes regions responsible for speech articulation, such as the left and right cerebellum, the right frontal cortex, and the right supplementary motor area [10].

Table 2 summarizes the EEG-tDCS studies in healthy and neurological populations. Among the six studies considered, five were conducted with healthy people [35,68,69,70,71], and one study with post-stroke nonfluent aphasics [72].

In the five studies with healthy participants, the behavioral task coupled with tDCS mainly involved executive function trainings related to impulsivity or inhibitory components [35,68,69,70,71]. Two studies applied bi-hemispheric tDCS with the anode over the right IFG and the cathode over the left IFG [35,69]. In the study by Hertenstein et al. [35], the authors also considered a reverse protocol with anodal tDCS over the left IFG and cathodal tDCS over the right IFG (see Table 2). In the remaining four studies, anodal tDCS was applied over the right IFG [68,70,71,72]. It should be noted that in three out of five studies, neither the active nor the sham condition had an impact on performance at the behavioral level [68,70,71]. However, in two of these studies [68,70], the absence of significance in behavioral performance was accompanied by a decrease in the P3 amplitude in the tDCS group. Cunillera et al. [69] reported a larger number of inhibitory responses in the anodal session compared to the sham condition but only in one task (i.e., Go/No-go task). As in the other studies [68,70], ERP results revealed a reduction in the P3 amplitude [69]. In the study by Hertenstein et al. [35], creative performance, together with a power increase in fast (beta) frequencies, increased only with activation of the right and deactivation of the left IFG (L-R+ group) [35].

In the only study involving neurological participants, anodal tDCS over the right IFG was associated with language training aimed at improving speech fluency in a group of aphasic individuals. After the treatment, a greater number of correct syllable and sentence production was observed in the tDCS condition compared to sham, which was accompanied by an increase in the amplitude of ERP [72].

## 4. Discussion

This review reports a systematic analysis of the literature related to the effects of tDCS over the IFG to enhance cognition in healthy individuals and neurological populations by measuring functional changes through neuroimaging or electrophysiological techniques.

As introduced earlier, the integration of neuroimaging and electrophysiological techniques in tDCS research has been pivotal in recent years [73]. Approaches such as fMRI and rs-fMRI have played a crucial role in understanding the neural mechanisms behind the cognitive enhancements facilitated by tDCS within specific brain regions and their associated networks [74]. Notably, while fMRI has unveiled the modulation effects on functional connectivity across various brain networks [60,61,62], rs-fMRI has provided deeper insights into the impact of tDCS on intrinsic brain activity, shedding light on the broader neuroplastic changes induced by the intervention [10,61]. Moreover, these methodologies have enabled researchers to make causal inferences regarding the relationships between brain activity and behavior based on the induced effects [75]. More recent studies have expanded this understanding by investigating changes in cortical activity induced by tDCS using fNIRS [58,59]. Electrophysiological methods, such as EEG and ERPs, have also provided insights into the immediate effects of tDCS on neural oscillations and event-related brain responses [76].

Nevertheless, while our comprehensive investigation yields promising results overall, one significant aspect warrants discussion: The absence of significant behavioral changes reported in certain studies despite observable alterations in neural connectivity [37,59,64,68,70,71]. Given that the objective of applying tDCS should consistently involve enhancing cognitive treatment outcomes as an adjunctive tool [7], this aspect merits careful consideration.

Due to the diversity of experimental designs employed, it is very difficult to come to an unequivocal understanding of these disparities. Nonetheless, one potential explanation is that the neural alterations observed in certain studies [37,59,64,68,70,71] may have occurred in regions or circuits not directly linked to the specific behavior under assessment, thus failing to reflect the impact of cognitive training. For instance, in the study by Li et al. [37], tDCS applied over the right IFG heightened connectivity within the default mode network, typically active during passive rest periods when individuals are not engaged in specific cognitive tasks [77]. Additionally, it is plausible that behavioral enhancements necessitate a certain threshold of neural change to become perceptible. Minimal or localized shifts in neural activity might not have surpassed this threshold, resulting in a lack of observable behavioral improvement. This rationale could elucidate the findings reported by Ehlis et al. [59] and Nissim et al. [64], wherein the activation of regions presumably involved in the cognitive task did not coincide with behavioral changes.

As noted in previous research [22,78,79], another crucial aspect to consider, which could contribute to a clearer interpretation of the inconsistencies observed in the aforementioned studies [37,55,59,64,65,66], is the variability in individual responses to tDCS, a factor overlooked by the authors as they analyzed results at the group level. Despite being frequently disregarded in many tDCS investigations, recent reports have highlighted that when individual responses to tDCS are analyzed, only half—or even fewer—of the participants exhibit the expected response (termed as good responders) [78,79]. Interestingly, discrepancies in participants’ anatomical brain structures, such as skull thickness, scalp-to-cortex distance, and cortex folding, appear to significantly influence result interpretation. Supporting this notion, modeling studies have indicated that individual anatomical variations may impact the distribution of electric fields across the stimulated cortex, subsequently influencing participant responses [78,79,80,81,82]. For instance, in a modeling study by Kim et al. [79], conduction current density values at the dorsolateral prefrontal cortex due to tDCS were notably higher in good responders compared to poor responders in a working memory task. Therefore, one might speculate that, in the aforementioned studies [37,59,64,68,70,71], any variances in participants’ responses to real and sham conditions could have been lessened by examining the data at the group level, while differences among participants still influenced the detected neural alterations.

Encouragingly, six fMRI studies performed in healthy participants showed significant variations both at the behavioral and the neural level following tDCS intervention over the left [58,60,61,62,63] or the right IFG [11]. Indeed, the behavioral improvement significantly correlated with a decrease in the functional activity of neural networks pertaining to the cognitive task performed [11,58,60,61,62,63]. In Fiori et al. [60], the improvement found in verb learning induced by anodal tDCS over the left IFG was related to an overall decrease in processing effort within a large language network, including the left IFG and the right homolog. Decreased task-related activity under anodal tDCS over the left IFG during language learning with more efficient cognitive processing was also observed in three other fMRI studies [61,62,63]. Accordingly, the neural efficiency hypothesis [83,84] claims that decreased brain activity in individuals with improved cognitive performance reflects greater efficiency in the task-specific neural network due to neuronal adaptation [61,85]. Notably, in two studies [61,62], increased functional connectivity between the stimulated left IFG and other key regions for language was found at rest, indicating that tDCS has modulated functional interactions on a larger network level. Such large-distance remote effects due to brain stimulation have been reported by some other studies in the motor and language domains [86,87,88]. In line with these findings, in the f-NIRS study by De Rosa et al. [58], increased hemodynamic activity during a working memory task was also present in the contralateral (with respect to stimulation) region. Thus, as in fMRI studies [60,61,62,63], this result confirms that tDCS modulates brain areas that are not necessarily located below the electrode itself. Indeed, in Sandrini et al. [11], anodal tDCS induced significant changes both behaviorally, on a response inhibition task, and in functional connectivity between the targeted right IFG and subcortical regions (e.g., caudate), as well as in other regions (e.g., right dorsolateral prefrontal cortex and right inferior parietal cortex, see also [89,90]) pertaining to the cognitive task assessed. Consistent with these findings, tDCS-fMRI studies performed in different neurological populations [10,57,65,66,67] also reported significant improvement in language tasks following left IFG stimulation, which was accompanied by neuronal changes across broad networks.

Thus, taken as a whole, these results suggest that the dynamic neural modulation exerted by tDCS is likely to influence not only spontaneous brain activity but also the strength of functional connectivity between interconnected network nodes, which, in turn, enhances task processing efficiency [57,61,63]. Therefore, these results validate earlier neuroimaging research [75], underscoring the importance of pinpointing these central nodes to maximize the neuronal impact of stimulation [57,61,62,63].

Six studies also stimulated the right IFG with concomitant EEG monitoring to provide real-time data on tDCS impact on cortical excitability while subjects performed a cognitive task [35,68,69,70,71,72]. As in the previous fMRI studies, the modifications reported at the neural level were not always accompanied by changes at the behavioral level [68,70,71]. One possibility for the absence of this significance might be that the task used, which principally involved response inhibition processes (Go/No-go task), was not challenging enough for the healthy participants to trigger behavioral modifications [68,70,71]. However, using the same task, Cunillera et al. [69] found behavioral changes using a bi-hemispheric tDCS montage with deactivation of the left and activation of the right IFG. Similarly, in Hertenstein et al. [35], creative performance increased by applying a bilateral montage. It has been well established that inhibitory control is implemented by specific fronto-basal-ganglia circuits, which involve both the right and the left IFG [91]. Studies from patients with damage in the left IFG indicated that the integrity of the left IFG is also critical for the successful implementation of inhibitory control over motor responses. Indeed, in the Swick et al. study [91], the spared RIFG was not sufficient to compensate for the effect of the LIFG lesion. Accordingly, functional neuroimaging studies have argued that, although the right hemisphere regions, particularly the right dorsolateral prefrontal cortex and the right IFG, are predominant for inhibitory control [92,93], the contribution of the left IFG should also be considered [94,95,96,97]. Thus, it could be the case that in these last two studies [35,69], the current, simultaneously delivered with excitation of the right and inhibition of the left IFG, acted more efficiently than when unilaterally applied over the right IFG [68,70,71] by reinforcing the activation in the predominant right IFG through inhibition of the left homologous. Interestingly, in all studies that employed a response inhibition paradigm [68,69,70,71], tDCS over the RIFG determined a decrease in the amplitude of the ERP3 component, which is a marker of the inhibitory function [98,99].

As far as we know, only one study investigated the electrophysiological correlates of tDCS over the IFG combined with cognitive training in a group of aphasic individuals [72]. In Cipollari et al. [72], language treatment alone (sham) was associated with cortical excitability changes in the transcranial magnetic evoked potentials (TEPs). Interestingly, the data also showed that these changes were maximized when the language treatment was associated with anodal tDCS over the right IFG, as confirmed by the modulation of the same TEPs components reaching their maximal amplitude during the post-anodal condition. Thus, anodal stimulation further increased the beneficial effects of the treatment. However, it must be noted that because of the limited number of patients, no correlation analysis was performed in this study to directly link the electrophysiological to the behavioral data [72]. Moreover, since the authors did not measure TEPs signals from other brain areas (e.g., the contralateral left IFG), it cannot be excluded that given the low spatial resolution of tDCS, the stimulation influenced cortical excitability in areas other than the targeted one [72].

Prior to drawing conclusions, it is crucial to recognize that all the mentioned studies underscored not only the effectiveness of tDCS in eliciting neural changes within localized brain areas and interconnected networks but also affirmed the significant role of the IFG across various cognitive tasks [26,27,28,30,32,33,34,100]. Indeed, it seems that regardless of the population under study (whether healthy or neurological), the left IFG plays a central role not only in language-related tasks but, more notably, when stimulated, it acts as a core node within an extensive language-dedicated network [60,61,62,63]. Similarly, the right IFG serves as a pivotal center for creative endeavors and tasks involving executive functions, leveraging its extensive connections with other brain regions [11,37], see also [89,90,92]. Thus, owing to its centrality in cognitive control circuits, employing tDCS to stimulate the IFG holds promise in enhancing neural activity within specific networks, thereby increasing performance across various domains such as working memory, decision-making, and language.

## 5. Conclusions

Although definitive conclusions cannot be drawn from the above-reported studies, certain aspects warrant thoughtful consideration. First, a consistent observation across most studies is the efficacy of targeting the IFG using tDCS to modulate extensive networks associated with the cognitive task. Thus, the IFG serves as a central hub that, when stimulated, enhances various cognitive aspects and promotes functional connectivity changes among interconnected regions. Specifically, the left IFG predominantly influences language tasks, while the right IFG appears more engaged in creative processes, problem-solving, and tasks requiring response inhibition and selective attention. Notably, this modulation does not necessarily result in a generic increase in task-related activity patterns. Instead, enhanced connectivity within each network seems to underlie improved neural efficiency in highly specific brain areas crucial for task execution. Consequently, considering that cerebral lesions typically induce irreversible neuronal tissue damage, tDCS’s capacity to influence large neural networks and its distant effects may potentially offer clinical benefits by more efficiently activating compensatory mechanisms.

In conclusion, we posit that tDCS stands to gain from insights obtained through brain mapping techniques. Neuroimaging and electrophysiology can refine tDCS parameters by offering valuable information about its mechanisms of action. Moreover, they may serve as more sensitive biomarkers to detect post-tDCS changes not yet discernible at the behavioral level.

## Figures and Tables

**Table 1:** tDCS studies combined with cognitive training and neuroimaging in healthy and neurological populations.

Authors	Healthy Participants	tDCS Protocol	Cognitive Training	Behavioral and Neuroimaging Outcomes
Di Rosa et al. 2019 [58]	N = 21 Mean Age (years) = 69.7 ± 5.05 Healthy People	Anode = LIFG Cathode= contralateral shoulder No. of sessions = 1 Duration = 26 min Current density 1.5 mA	Visuo-spatial WM task	- In the WM task, faster responses were observed in the anodal tDCS condition, but no differences were found in the sham condition. - Higher HbO activity was detected through fNIRS in the bilateral frontal regions during anodal tDCS.
Ehlis et al., 2016 [59]	N = 46 Mean Age (years)= 32.1 ± 10.5 (N = 23 anode LIFG) Mean Age (years)= 24.3 ± 2.4 (N = 23 cathode LIFG) Healthy People	Anode or Cathode = LIFG Reference electrode = contralateral shoulder No. of sessions = 1 Duration = 26 min Current density 1.5 mA	Verbal fluency task (VFT)	- Brain activation was measured by fNIRS during task performance. Neither anodal nor cathodal tDCS was found to modulate VFT performance. However, anodal tDCS increased brain activity in the prefrontal cortex during the VFT.
Fiori et al., 2019 [60]	N = 28 Mean Age (years) = 26.96 Healthy People	Anode = LIFG Cathode = contralateral fronto-polar region No. of sessions = 1 Duration = 24 min Current density 1 mA	Verb learning task	- A-tDCS significantly decreased task-related activity at the stimulated left IFG, in the right homolog, and between the left IFG and the right insula. - The individual decrease in connectivity was significantly correlated with individual behavioral improvement during A-tDCS.
Holland et al., 2011 [61]	N = 10 Mean Age (years) = 69 Healthy People	Anode = LlFG Cathode = right frontopolar cortex No. of sessions = 1 Duration = 20 min Current density 2 mA	Picture naming task	- Faster picture naming performance in A-tDCS compared to sham. - Faster naming responses were associated with a decrease in the BOLD signal detected through fMRI in the LIFC in A-tDCS compared to sham.
Li et al., 2019 [37]	N = 26 Mean Age/SD (years) = 38 ± 15.5 Healthy People	Anode/Cathode = RIFG Cathode/Anode = right shoulder No. of sessions = 3 Duration = 18 min Current density 1.8 mA	Choice reaction task (CRT)	- There were no significant effects of stimulation on behavioral performance. - Cathodal tDCS accentuated the within-DMN connectivity detected through fMRI associated with task performance.
Meinzer et al., 2012 [62]	N = 20 Mean Age/SD (years) = 26.7 ± 3.8 Healthy People	Anode = LIFG Cathode = contralateral supraorbital region No. of sessions = 1 Duration = 18 min Current density 1 mA	Semantic word generation task	- Improved word retrieval during A-tDCS was paralleled by selectively reduced task-related activation in the left IFG, an area specifically implicated in semantic retrieval processes. - Rs-fMRI revealed increased connectivity in the LIFG and other major hubs overlapping with the bilateral language network during A-tDCS compared with sham. - The cluster with the strongest tDCS-induced increase was found in the LIFG and anterior insula. Additional significant clusters were in the bilateral inferior parietal, dorsolateral, medial prefrontal regions, and the left middle temporal gyrus.
Meinzer et al., 2013 [63]	N = 20 Mean Age/SD (years) = 68.0 ± 5.7 Healthy People	Anode = LIFG Cathode = contralateral supraorbital region No. of sessions = 1 Duration = 20 min Current density 1 mA	Semantic word generation task	- Significantly more correct responses during A-tDCS compared with sham. - Task-related fMRI analysis showed significantly reduced activity in bilateral IFG and RMFG in the A-tDCS condition compared to sham. - Improved performance during A-tDCS was associated with reduced activity in RMFG.
Nissim et al., 2019 [64]	N = 16 Mean Age/SD (years) = 71.75. ± 7.29 Healthy People	Anode = LIFG Cathode = RIFG No. of sessions = 1 Duration = 12 min Current density 2 mA	Working memory task	- Percent accuracy and reaction time on the N-Back task did not significantly differ between active and sham stimulation conditions at the various time points. - Active tDCS produced a significant increase in functional connectivity between the left VLPFC and the left DLPFC corresponding to the working memory network. - Connectivity did not significantly increase with sham stimulation.
Sandrini et al., 2020 [11]	N = 26, SD = ±4; (active group) N = 27, SD = ±6 (sham group) Healthy People	Anode = RIFG Cathode = contralateral supraorbital region No. of sessions = 1 Duration = 20 min Current density 1.5 mA	Stop signal response time (SSRT)	- A-tDCS facilitated response inhibition relative to sham also modulating functional connecftivity in the fronto-basal ganglia as well as RDLPFC and RIPC as an integral part of the response inhibition network.
Das et al., 2019 [65]	N = 22 Mean Age/SD (years) = 62.91 ± 7.79 Mild Cognitive Impairment (MCI)	Anode = LIFG Cathode= contralateral shoulder No. of sessions = 8 Duration = 20 min Current density 2 mA	SMART training	- Only the sham condition showed significant immediate cognitive gains in executive functions and episodic memory tasks. - A significantly larger increase in CBF detected through fMRI was observed in the RMFG in A-tDCS + SMART compared to S-tDCS + SMART.
Meinzer et al., 2015 [57]	N = 18 Mean Age/SD (years) = 67.44 ± 7.27 MCI	Anode = LIFG Cathode = contralateral supraorbital region No. of sessions = 1 Duration = 20 min Current density 1 mA	Semantic word retrieval task	- A-tDCS significantly improved semantic word retrieval performance. - Reduced activity in the LIFG, LDLPC, RIFG, and RMFG was detected through fMRI during A-tDCS compared to sham. - A-tDCS compared with sham resulted in increased connectivity bilaterally in lateral and medial frontal and sensorimotor cortices and left occipito-temporal regions in rs-fMRI.
Ficek et al., 2018 [66]	N = 24 Mean Age/SD (years) = 67.2 ± 6.5 Primary Progressive Aphasia (PPA)	Anode = LIFG Cathode = right cheek No. of sessions = 15 Duration = 20 min Current density 2 mA	Written language training	- Both the tDCS and sham groups showed significant improvements in the percentage of trained correct letters after the treatment. - Significant association between the behavioral improvement (letter accuracy) and connectivity changes measured through fMRI in the left IFG triangularis and left ITG. - Significant negative tDCS effect on FC between left IFG orbitalis and left MTG.
Tao et al., 2021 [67]	N = 32 Mean Age/SD (years) = 67 ± 6.73 PPA	Anode = LIFG Cathode = right cheek No. of sessions = 15 Duration = 20 min Current density 2 mA	Written language training	- At the immediate post-treatment time-point, tDCS showed a significant augmentative effect in behavioral outcome (letter accuracy) over sham, which was maintained at a 2-month follow-up. - The tDCS group showed a significant decrease in overall connectivity, while the sham group did not change; the decrease was due to reduced connectivity between LIFG and regions outside the perisylvian language area.
Marangolo et al., 2016 [10]	N = 9 Age (years) = 47–70 Nonfluent aphasic patients	Anode = LIFG Cathode = RIFG No. of sessions = 15 Duration = 20 min Current density 2 mA	Repetition task	- The mean percentage of response accuracy significantly improved only in the tDCS condition. - tDCS effects revealed that EC increased in the left and right cerebellum, in the left premotor cortex, in the left ACC, in the LMFG, in the left precuneus, in the right frontal cortex, and in the right supplementary motor area. - A significant correlation was found between EC increase and syllable repetition accuracy changes.

Legend: LPFC: Left Prefrontal Cortex; fNIRS: Functional Near-Infrared Spectroscopy; WM: Working Memory; HbO: Oxy-Hemoglobin Concentration; LIFG: Left Inferior Frontal Gyrus; RIFG: Right Inferior Frontal Gyrus; DMN: Default Mode Network; RsfMRI: Resting-State Functional Magnetic Resonance; RMFG: Right Middle Frontal Gyrus; VLPFC: Ventro-Lateral Prefrontal Cortex; LDLPFC: Left Dorsolateral Prefrontal Cortex; RDLPFC: Right Dorsolateral Prefrontal Cortex: RIPC: Right Inferior Parietal Cortex; SMART: Strategic Memory and Advanced Reasoning Training; CBF: Cerebral Blood Flow; LITG: Left Inferior Temporal Gyrus; FC: Functional Connectivity; LMTG: Left Middle Temporal Gyrus; EC: Eigenvector Centrality (topological measure); ACC: Anterior Cingulate Cortex.

**Table 2:** tDCS studies combined with cognitive training and EEG in healthy and neurological populations.

Authors	Healthy Participants	tDCS Protocol	Intervention Task	Behavioral and EEG Outcomes
Campanella et al., 2016 [68]	N = 31 (15 active group/16 sham group) Mean Age/SD (years) = 21.9 ± 3.1 Healthy People	Anode = RIFG Cathode = the neck No. of sessions = 1 Duration = 20 min Current density 1 mA	Go/No-go task	- Neither tDCS nor sham had an impact on performance at the behavioral level. - Decreased P3d amplitude for the Go/No-go task in the tDCS group, indicating less recruitment of neural resources to perform the task correctly.
Cunillera et al., 2016 [69]	N = 13 Mean Age/SD (years) = 25.2 ± 3.3 Healthy People	Anode = RIFG Cathode = LIFG No. of sessions = 1 Duration = 20 min Current density 1.5 mA	Go/No-go task Stop signal reaction time (SSRT)	- The participants inhibited their responses in a significantly larger number of trials in the anodal session compared to the sham tDCS session in the Go/No-go task. - The ANOVA results revealed a non-significant effect for both tDCS sessions in the SSRT task. - ERP results revealed that tDCS reduced the amplitude of the inhibitory-P3 in NoGo and Stop correct inhibited trials.
Hertenstein et al., 2019 [35]	N = 90 Mean Age/SD (years) = 23.8 ± 2.3 Healthy People	Anode = LIFG/RIFG Cathode = RIFG/LIFG No. of sessions = 1 Duration = 22 min Current density 1 mA	Alternate uses task (AUT), Compound remote associate task (CRA), Wisconsin card sorting task (WCST)	- Creative performance increased with deactivation of the left IFG and activation of the right IFG (L-R+ group) and reduced with the reverse protocol (L + R-group). - Resting state EEG analyses indicated increased neural excitability after anodal tDCS over the RIFG indexed by a power increase in fast (beta) frequencies.
Mendes et al., 2024 [70]	N = 40 Mean Age/SD (years) = 23.2 ± 3.52 Healthy People	Anode = RIFG Cathode = left mastoid No. of sessions = 2 (1 active and 1 sham tDCS) Duration = 20 min Current density 2 mA	Waiting impulsivity task (CPRT) Stop signal reaction time (SSRT)	- No modulatory effects of tDCS over rIFG were found in terms of waiting impulsivity and inhibitory control measures (i.e., CPRT and SSRTT, respectively). - Anodal tDCS decreased the target-P3 amplitude and underlying oscillatory activity (delta power) during the waiting impulsivity task.
Thunberg et al., 2020 [71]	N = 18 Mean Age (years) = 24 Healthy People	Anode = right IFG Cathode = visual cortices No. of sessions = 3 Duration = 20 min Current density 2 mA	Stop signal task (SST) Stop signal reaction time (SSRT)	- Neither condition was associated with changes in SSRTs and in stop signal delays. - tDCS did not affect goRTs. - tDCS did not modulate P3 peak latencies.
Cipollari et al., 2015 [72]	N = 6 Mean Age (years) = 59.16 post-stroke nonfluent aphasic patients	Anode= RIFG Cathode = contralateral fronto-polar cortex No. of sessions = 15 Duration = 20 min Current density 2 mA	Melodic intonation therapy (MIT)	- At the end of treatment, the percentage of correct sentence repetition was significantly higher in the active than in the sham condition. - Amplitude of the TEPs increased after anodal tDCS when compared to the baseline and the post-sham sessions.

Legend: NT: No Training; IT: Inhibition Training; RDLPFC: Right Dorsolateral Prefrontal Cortex; R/LIFG: Right/Left Inferior Frontal Gyrus; TEPs: Transcranial Magnetic Evoked Potentials.

## Data Availability

Not applicable.
